# Editorial: Getting to the heart of developmental toxicities

**DOI:** 10.3389/ftox.2023.1138470

**Published:** 2023-01-16

**Authors:** Elizabeth C. Bowdridge, Jennifer Thompson, Stephane Bourque, Phoebe Stapleton

**Affiliations:** ^1^ Department of Physiology, Pharmacology and Toxicology, West Virginia University School of Medicine, Morgantown, WV, United States; ^2^ Department of Physiology and Pharmacology, University of Calgary, Calgary, AB, Canada; ^3^ Department of Anesthesiology and Pain Medicine, University of Alberta, Edmonton, AB, Canada; ^4^ Department of Pharmacology and Toxicology, Ernest Mario School of Pharmacy, Rutgers University, Piscataway, NJ, United States

**Keywords:** reproduction, DOHaD (development origins of health and disease), cardiovascular, development, placenta

The Developmental Origins of Health and Disease (DOHaD) hypothesis proposes that environmental exposures, particularly during gestation, can predispose offspring to chronic diseases and other poor health outcomes that persist into adulthood (Barker, 2007). During gestation the fetus is dependent upon the blood flow from the dam to provide the proper delivery of nutrients and oxygen for growth, as well as removal of fetal waste products. The source of maternal-fetal blood flow may be described as originating in the maternal heart and therefore any exposure that alters its function has the capability to disturb uteroplacental hemodynamics as well as fetal viability and growth, with potential lasting subsequent cardiometabolic consequences throughout life. Specifically, gestational environmental exposures can lead to cardiovascular disease, and/or complications from impacts on the fetal cardiovascular system such as renal or metabolic dysfunction. The interface between the mother and baby is the transient, but critically important, placenta. The relationships between maternal environmental influences, cardiovascular function, and fetal health is a budding field. This Research Topic contains three original research papers and a review that spans both rodents and humans highlighting the broad applicability of this topic and its importance to toxicology.

D'Errico et al. discuss their findings on fetal growth restriction (FGR) based on intrauterine location after exposure to the engineered nanomaterial, nano-titanium dioxide (nano-TiO_2_). Unlike humans, rodents are multiparous and therefore the location of the pup within the uterus may affect its growth trajectory (McLaurin and Mactutus, 2015). Using a Sprague Dawley rat model, the authors found that exposure throughout gestation did not affect placental weight, but fetal weights were impacted based on intrauterine location (i.e., left/right horn; ovarian, middle, cervical implantation). These outcomes were exacerbated after nano-TiO_2_ exposure and gestational day that exposures were initiated within the dam. The findings of this paper should encourage other studies to consider intrauterine position, maternal weight, and the number of fetuses per horn to obtain a more sensitive evaluation of FGF in rodent models.

Bisphenol A (BPA) is the most commonly used bisphenol in polycarbonate plastics and epoxy resins. A multitude of studies have shown that BPA acts as an endocrine disruptor, and therefore, many companies are replacing BPA with similar structural analogues such as bisphenol S (BPS). This is problematic considering there is mounting evidence that BPS can also act as an EDC due to its estrogenic properties (Connors et al.). showed that prenatal exposure to BPS, even at a low dose, leads to sexually dimorphic outcomes relating to cardiovascular responsivities such that only female vascular sensitivities identified may be associated with altered nitric oxide and estrogen signaling. This work underscores the importance of sex as a variable in studies investigating EDC exposure during gestation.

Mandakh et al. evaluate a population of women in Sweden to study the association of ambient air pollution with the development of preeclampsia (PE). In addition, the authors investigated if this association was a result of oxidative stress within the placenta leading to changes in telomere length and mitochondrial DNA copy number (mtDNAcn). They identified that exposure to high levels of ambient nitrogen oxides (NO_X_, a marker for traffic related air pollution) during the first trimester or throughout pregnancy was associated with reduced relative placental mtDNAcn and increased risk of PE. However, there was no data to support mtDNAcn or telomere length as the mediators of this association between air pollution and PE. The reduction in mtDNAcn does beg the question as to the role of mtDNAcn in other pregnancy complications related to air pollution during pregnancy. This study specifically brought a translational perspective to how fetomaternal health can be affected by environmental exposures and the complexities of population studies.

Lastly, the review by (Singh et al.) describe the potential link between prenatal exposure to endocrine disrupting chemicals (EDC) and what is now known as “cardiometabolic syndrome”, formerly known simply as “metabolic syndrome or syndrome X”. The authors discuss how gestational exposure to EDCs during fetal development may involve critical changes in the hormonal and epigenetic imprints within the offspring that predispose them to cardio-metabolic-renal (CMR) associated diseases later in life. The authors propose that the mechanism linking EDC exposure to poor adult CMR-health is an increased baseline inflammatory state and altered adipocyte development in the offspring. The authors specifically call for further studies on the imprinted phenotype resulting from EDC exposure during development affects CMR-health later in life. Identifying these epigenetic imprints may also allow for early or personalized treatments for individuals predisposed to CMR disease.

In conclusion, the contributions of the four articles to this Research Topic, “Getting to the Heart of Developmental Toxicities” exemplifies how gestational exposures can perturb both maternal and fetal cardiovascular health and function. Exposures to nanoparticles, EDCs, or ambient air pollution during critical developmental windows can result in poor fetal health outcomes that then persist into adulthood through lasting imprinted phenotypes. This collection of excellent science showcases a few avenues open to explore in the fields of cardiovascular function, fetal development, environmental exposures, and their intersections.
